# Identification of a novel protein promoting the colonization and survival of *Finegoldia magna*, a bacterial commensal and opportunistic pathogen

**DOI:** 10.1111/j.1365-2958.2008.06439.x

**Published:** 2008-09-25

**Authors:** Inga-Maria Frick, Christofer Karlsson, Matthias Mörgelin, Anders I Olin, Radmila Janjusevic, Clara Hammarström, Elisabet Holst, Maarten de Château, Lars Björck

**Affiliations:** 1 Department of Clinical Sciences, Division of Infection Medicine, Lund UniversitySE-22184 Lund, Sweden; 2 Department of Laboratory Medicine, Division of Medical Microbiology, Lund UniversitySE-22362 Lund, Sweden

## Abstract

Anaerobic bacteria dominate the human normal microbiota, but strikingly little is known about these commensals. *Finegoldia magna* is a Gram-positive anaerobe found in the skin and at other non-sterile body surfaces, but it is also an opportunistic pathogen. This study describes a novel protein designated FAF (*F. magna* adhesion factor) and expressed by more than 90% of *F. magna* isolates. The protein is present in substantial quantities at the *F. magna* surface but is also released from the surface. FAF forms large protein aggregates in solution and surface-associated FAF causes bacterial clumping. In skin *F. magna* bacteria were localized to the epidermis, where they adhere to basement membranes. FAF was found to mediate this adhesion via interactions with BM-40, a basement membrane protein. The biological significance of FAF is further underlined by the observation that it blocks the activity of LL-37, a major human antibacterial peptide. Altogether, the data demonstrate that FAF plays an important role in colonization and survival of *F. magna* in the human host.

## Introduction

In the normal human microbiota that colonizes the skin and mucous membranes anaerobic bacterial species dominate, especially in the gastrointestinal tract where more than 90% of the bacterial population consist of obligate anaerobes (Tlaskalová-Hogenová *et al*., 2004). The abundance and significance of anaerobic bacteria, in the normal microbiota and in clinical infections, are probably highly underestimated because of problems obtaining good quality anaerobic specimens, and experimental difficulties connected with the growing and handling of these bacteria. The Gram-positive coccus *Finegoldia magna* (formerly *Peptostreptococcus magnus*) is one of the most ubiquitous anaerobic species in the skin (Higaki and Morohashi, 2003), but it also inhabits the oral cavity and the gastrointestinal and urogenital tracts (Murdoch, 1998). Apart from being a commensal *F. magna* is also an opportunistic pathogen, and among the anaerobic bacteria of the normal microbiota, *F. magna* is the species most frequently isolated in pure culture from patients with clinical infections. Typical infections connected with *F. magna* are soft tissue abscesses, bone/joint infections, wound infections and vaginosis (Murdoch, 1998; Stephens *et al*., 2003). Rare cases of prosthetic valve endocarditis caused by *F. magna* have also been reported (van der Vorm *et al*., 2000; Bassetti *et al*., 2003).

Possible *F. magna* pathogenicity factors have been identified, including capsule formation (Brook, 1986) and production of various enzymes, such as collagenase and gelatinase (Krepel *et al*., 1992). Most strains express a subtilisin-like enzyme, SufA, showing proteolytic activity against antimicrobial molecules like LL-37 and MIG/CXCL9 (Karlsson *et al*., 2007). Approximately 10% of *F. magna* isolates express a surface protein, protein L, with affinity for immunoglobulin light chains (Björck, 1988), whereas the majority of *F. magna* isolates from patients with bacterial vaginosis expresses protein L (Kastern *et al*., 1990). A role for protein L in *F. magna* pathogenicity is also indicated by the finding that when expressed atthe surface of *Streptococcus gordonii* bacteria, protein L enhances their ability to colonize the vaginal mucosa of mice (Ricci *et al*., 2001). Another surface protein of *F. magna*, the albumin-binding protein PAB (de Château and Björck, 1994) is commonly expressed by isolates from localized suppurative infections, such as deep wound infections (de Château *et al*., 1996), suggesting that PAB could also contribute to the pathogenicity of *F. magna*.

Very limited information is available concerning the molecular mechanisms that promote the colonization and survival of anaerobic commensals in the skin and at other non-sterile sites. In the present work we describe the properties of a novel protein of *F. magna*, with an impact on some basic prerequisites for the relationship between this commensal and its human host.

## Results

### Surface protein of *F. magna* induces bacterial aggregation

**Fig. 1:**
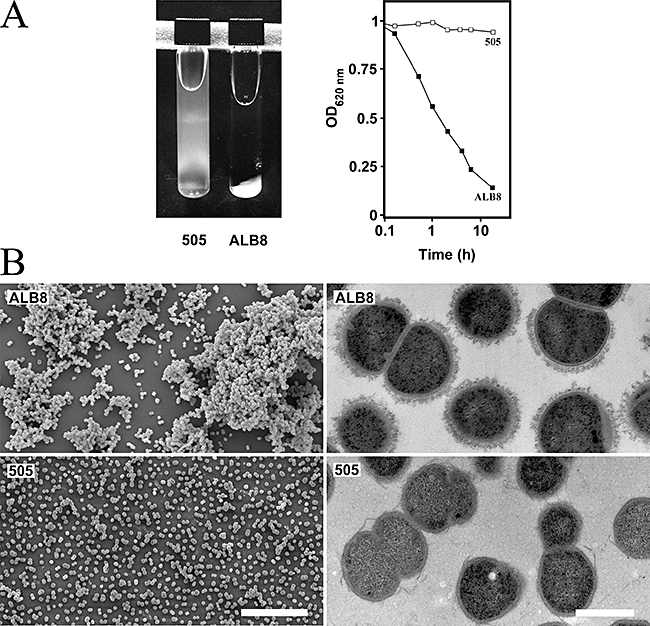
Analysis of *F. magna* aggregation. A. *F. magna* strains ALB8 (▪) and 505 (□) were grown under strict anaerobic conditions at 37°C for 3 days in TH broth supplemented with 0.5% Tween-80. Bacteria were resuspended and left to settle at 4°C. The optical density (620 nm) in the upper half of the test tubes was measured at various time points. B. Left panel: scanning electron micrographs showing ALB8 and 505 bacteria from 3 days cultures in TH broth. The bar represents 10 μm. Right panel: Transmission electron micrographs of the same cultures. The bar represents 0.5 μm.

**Fig. 2:**
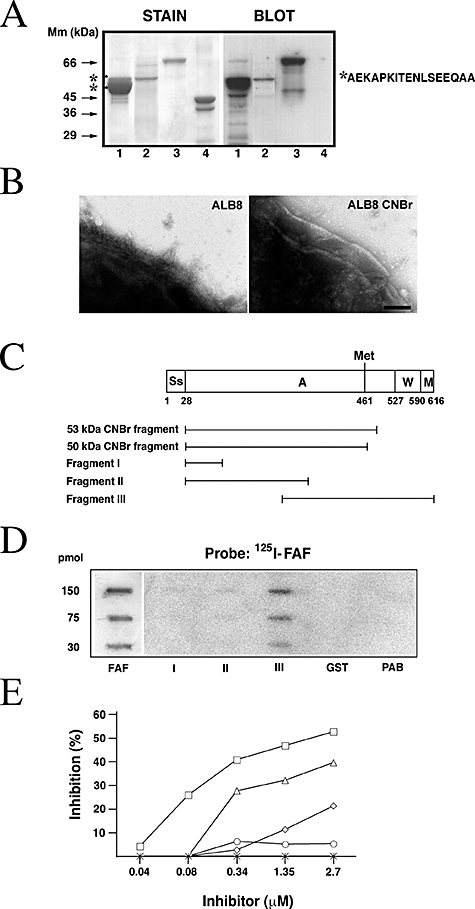
Protein FAF self-associates. A. Solubilized surface proteins from *F. magna* ALB8 bacteria were separated by SDS-PAGE. Two identical gels (10%) were run simultaneously; one was stained with Coomassie blue (STAIN), and one was blotted onto a PVDF membrane and probed with ^125^I-labelled recombinant FAF (BLOT). Lane 1: proteins released with CNBr; lane 2: proteins shedded from the surface of ALB8 bacteria and purified as described (see *Experimental procedures*); lane 3: recombinant FAF; lane 4: recombinant protein PAB. Bands indicated in the figure with a star were excised and subjected to N-terminal amino acid sequencing. B. Electron micrographs after negative staining of untreated and CNBr-treated ALB8 bacteria. The bar represents 100 nm. C. Schematic representation of FAF. The signal sequence (Ss), the alanine rich region (A), the methionine residue (Met), the wall spanning (W) and the membrane spanning (M) regions are indicated. The fragment obtained by CNBr treatment and the recombinantly expressed fragments of FAF are shown below. Numbers refer to amino acid residue positions. D. Different amounts of FAF, fragments of FAF, protein PAB and GST were applied onto a PVDF membrane. The membrane was incubated with ^125^I-labelled FAF, and binding was detected using the Fuji Imaging System. E. The binding of ^125^I-labelled FAF to ALB8 bacteria at a concentration of 2 × 10^9^ cfu ml^−1^ was inhibited with various amounts of unlabelled intact FAF (□), fragment I (◊), fragment II (○) or fragment III (▵) of FAF (see 2C) or protein PAB (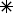).

When grown in liquid medium most clinical isolates of *F. magna* form visible aggregates. The starting point for the present work was the question how this property influences the biology of *F. magna* and how to explain the molecular basis for the aggregation. In contrast to a non-aggregating isolate (strain 505), bacteria of the ALB8 strain rapidly form large aggregates in the test tube (Fig. 1A). Analysis of the bacterial cultures by electron microscopy confirmed this observation (Fig. 1B, left panel) and revealed hair-like surface projections on ALB8 bacteria, mediating interactions between neighbouring bacterial cells. Such projections were not present at the surface of 505 bacteria (Fig. 1B, right panel). Following treatment of ALB8 with proteases or cyanogen bromide (CNBr) that removed the surface projections (Fig. 2B), the bacteria no longer formed aggregates suggesting that surface proteins play a role in bacterial clumping. CNBr cleavage released two quantitatively dominating protein fragments of apparently 50 and 53 kDa respectively, as determined by SDS-PAGE analysis (Fig. 2A, STAIN, lane 1). These were subjected to N-terminal amino acid sequencing analysis, and identical sequences were obtained from both bands (AEKAPKITENLSEEQAA), suggesting that they represent fragments of the same protein. When the database was searched, no sequences related to the obtained peptide sequence were found. Subsequent work described below showed that this novel protein promotes the adhesion and colonization of *F. magna*. It was therefore designated protein FAF (*F. magna* adhesion factor).

### Protein FAF is a self-associating protein

For cloning of the gene encoding protein FAF, a primer was constructed based on the N-terminal sequence described above. Together with a primer based on the cell wall-spanning LPXTG motif, a sequence known to be conserved among Gram-positive surface proteins (Schneewind *et al*., 1993), a PCR product of 1.7 kb was generated with chromosomal DNA of *F. magna* strain ALB8 as the template. The PCR product was purified, cloned and sequenced (see *Experimental procedures*). Analysis of the protein sequence deduced from the nucleotide sequence (GenBank accession number AY192570) shows a pre-protein of 616 amino acids with a predicted signal sequence of 27 amino acid residues (see schematic representation Fig. 2C). The mature protein of 589 amino acids has a molecular mass of 64.7 kDa. InterPro domain searches (Quevillon *et al*., 2005) revealed that FAF in its C-terminal part has a cell wall-spanning region, a membrane anchor and an intracellular charged tail typical of surface proteins of Gram-positive bacteria. The sequence contains no internal repeats but has high alanine content, especially in the N-terminal half. The C-terminal region also contains a Cna protein B-type domain, found in *Staphylococcus aureus* collagen-binding protein (Deivanayagam *et al*., 2000).

If protein FAF is responsible for the aggregation of ALB8 bacteria, FAF should be able to self-associate. To address this question the protein was expressed in *Escherichia coli* in fusion with glutathione S-transferase (GST). The GST-tag was removed, and recombinant FAF was used in various binding studies. The size of the 50 kDa fragment generated by CNBr cleavage of ALB8 bacteria (Fig. 2A, lane 1) corresponds well to the methionine residue at position 461 in the sequence of protein FAF (Fig. 2C). In contrast, the 53 kDa band most likely represents a fragment that is spontaneously shedded from the bacterial surface as substantial amounts of such a FAF fragment are found in ALB8 growth medium (Fig. 2A, lane 2). Radiolabelled protein FAF interacts with both the 50 and the 53 kDa fragments, as well as with the full-length recombinant molecule (Fig. 2A, BLOT). In contrast, the albumin-binding protein PAB, which is also expressed at the surface of ALB8 bacteria (de Château and Björck, 1994), did not bind the radiolabelled probe (Fig. 2A, lane 4).

To map the self-associating region of FAF, fragments of the protein were constructed and recombinantly expressed in fusion with GST (Fig. 2C). The various FAF fragments and protein PAB were applied onto a polyvinylidene difluoride (PVDF) membrane and probed with ^125^I-FAF. Fragment III, covering the C-terminal part and full-length FAF, clearly bound the probe, while a weak binding occurred to the N-terminal fragments I and II (Fig. 2D). Protein PAB and the GST-tag alone did not bind ^125^I-FAF. In competitive binding experiments the full-length molecule and fragment III efficiently blocked the binding of ^125^I-labelled FAF to ALB8 bacteria. A partial inhibition was seen with the N-terminal fragment I (Fig. 2E). Despite a weak binding to FAF in slot blot, fragment II did not inhibit the binding of radiolabelled FAF to ALB8 (Fig. 2E). This discrepancy probably reflects that different affinities are required for a positive result and/or that the binding epitopes are differently exposed in the two experimental systems. Again protein PAB had no affinity for FAF. Taken together the results demonstrate that FAF, through self-association, contributes to the bacterial aggregation. Most likely, these interactions mainly occur in the C-terminal half of the molecule. Finally, antibodies raised against recombinant FAF further enhanced ALB8 sedimentation (data not shown), which underlines the importance of FAF in mediating bacterial aggregation.

### Secondary structure analysis of protein FAF

**Fig. 3:**
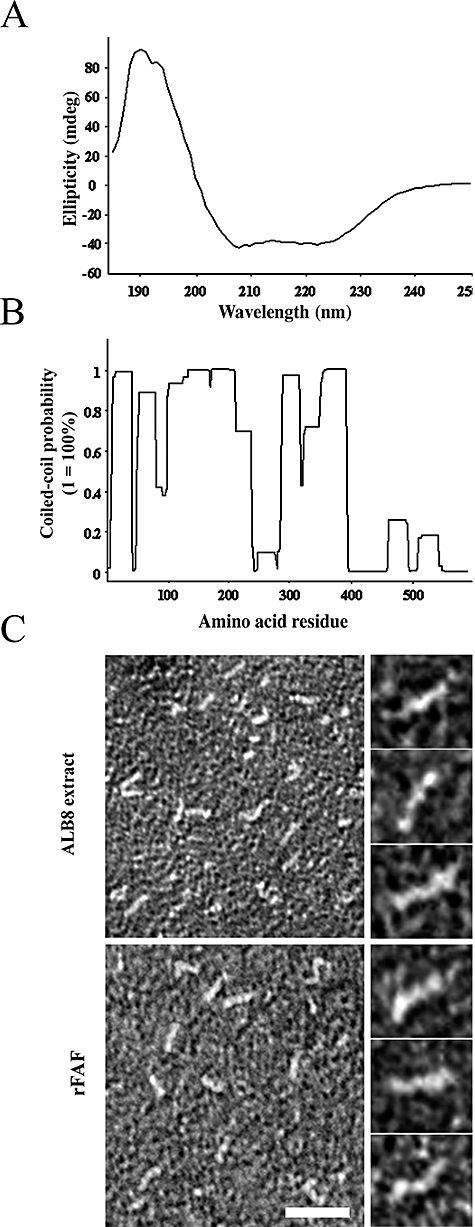
Secondary structure of protein FAF. A. Recombinant intact FAF at a concentration of 0.2 mg ml^−1^ was analysed by CD spectroscopy. The far UV spectral region at 20°C is shown. B. Coiled-coil prediction of FAF according to (Lupas *et al*., 1991) using the Macstripe program showing the probability on the *y*-axis and amino acid residue numbers on the *x*-axis. C. Electron micrographs after negative staining of FAF shedded from ALB8 bacteria (ALB8 extract) or recombinant FAF (rFAF). The bar represents 100 nm (left panel). Right panel: selected particles shown at higher magnification (bar = 50 nm).

Circular dichroism (CD) spectroscopy was used to analyse the secondary structure of FAF. Far UV spectra recorded at 20°C revealed that the structure of the molecule is predominantly α-helical, as indicated by the maximum below 200 nm and the double minima at 208 and 222 nm (Fig. 3A). Predictive secondary structure analysis according to the algorithm of Chou-Fasman and Robson-Garnier showed that most of FAF exhibits a strong α-helical potential. Analysis with the coiled-coil prediction algorithm of Lupas *et al*. in Macstripe (Lupas *et al*., 1991) yielded high coiled-coil forming probabilities in the major extracellular part (amino acids 28–417) of the molecule. The probability curve depicted in Fig. 3B indicates that some short regions in the N-terminal part, the C-terminal part of the A domain and the cell wall- and transmembrane-spanning regions of FAF do not adopt a coiled-coil structure. The secondary structure of the protein is similar to the α-helical coiled-coil structure of *Streptococcus pyogenes* M proteins (Phillips *et al*., 1981; Fischetti, 1989; Nilson *et al*., 1995). Protein FAF spontaneously released from ALB8 bacteria and the recombinantly expressed FAF were further analysed by electron microscopy after negative staining. The rod-like structure of the molecule fits well with a coiled-coil α-helical structure, and no difference could be observed between the two preparations (Fig. 3C).

### Distribution of the *faf* gene in *F. magna* strains

To investigate the expression of protein FAF in isolates of *F. magna,* 30 strains, including ALB8, were tested for binding of radiolabelled FAF. Among these, two were normal skin isolates and the others were from different clinical infections: urethritis, infected skin wounds, abscesses and vaginosis (see *Experimental procedures*). Binding varied between 9% and 28% of added radioactivity as compared with a background binding of 7% to 505 bacteria that lacks protein FAF. Surprisingly, PCR analyses of these isolates using oligonucleotides corresponding to the 5′- and the 3′-ends of the *faf* gene (faf1 and faf2) only generated the expected fragment of 1700 bp in a few strains (13.8%). However, when primers corresponding to other regions of the gene were used, 28 of the 30 strains were found to carry the gene (see *Experimental procedures*). The results indicate a sequence variation of FAF, mainly in the N-terminal part.

**Fig. 4:**
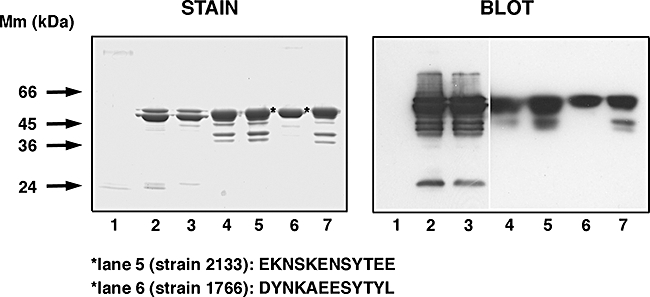
Analysis of FAF in seven *F. magna* strains. Proteins released by CNBr from the isolates were separated by SDS-PAGE. Two identical gels (10%) were run simultaneously; one was stained with Coomassie blue (STAIN), and one was blotted onto a PVDF membrane and probed with antibodies against FAF. Lane 1: strain 505; lane 2: strain ALB8; lane 3: strain L3410; lane 4: strain 1462; lane 5: strain 2133; lane 6: strain 1766; lane 7: strain ELTI. Bands indicated in the figure with a star were excised and subjected to N-terminal amino acid sequencing.

Five of the strains containing the *faf* gene (L3410, 1462, 2133, ELTI and 1766) were subjected to CNBr cleavage followed by SDS-PAGE analysis of released material. Similar fragments as those obtained for ALB8 were obtained from all strains (Fig. 4, STAIN). In Western blot the quantitatively dominating fragments of 50 and 53 kDa, released from all tested strains, reacted with antibodies raised against FAF from ALB8 bacteria, suggesting that homologous epitopes are present in these protein fragments (Fig. 4, BLOT). Radiolabelled FAF also bound to the fragments, supporting the presence of FAF homologues (data not shown). The dominating 50 kDa bands from strains 2133 and 1766 were subjected to N-terminal amino acid sequencing and the obtained sequences (2133: EKNSKENSYTEE; 1766: DYNKAEESYTYL) differed to that of ALB8. The *faf* genes of the *F. magna* strains L3410, 1462, 2133, ELTI and 1766 were sequenced, and the derived amino acid sequences were compared with that of FAF from strain ALB8 (Fig. S1). Strain L3410 expresses a protein almost identical to FAF of ALB8 (99% similarity), and the other strains express FAF homologues showing an overall sequence similarity of around 50% to ALB8 FAF (Fig. S1). There is a higher variability in the N-terminal regions, while the C-terminal halves are more conserved. Interestingly, FAF from strains 2133 and 1462 are identical, and strain ELTI has a FAF homologue closely related to these (94.1% similarity). Furthermore, in the complete genome sequence of *F. magna* ATCC 29328 strain recently reported (Goto *et al*., 2008), there is a FAF homologue displaying an overall sequence similarity of 75% to ALB8 FAF with a higher similarity in their C-terminal parts (89%) as compared with the N-terminal regions (60%) (Fig. S1). The data demonstrate that protein FAF variants are present at the surface of most *F. magna* strains.

### FAF interacts with the basement membrane protein BM-40

**Fig. 5:**
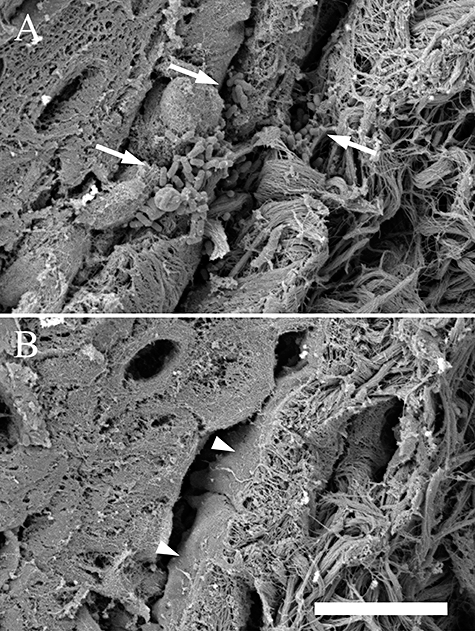
*F. magna* bacteria adhere to basement membranes. Human skin biopsies were incubated with ALB8 or 505 bacteria for 1 h at room temperature. Non-adherent bacteria were removed by washing in PBS, and the biopsies were incubated anaerobically at 37°C for 48 h, washed with PBST, fixed and prepared for SEM. A. ALB8 bacteria; B. 505 bacteria. The arrows point at ALB8 colonies and the arrowheads point at the basement membrane covering the junction between epidermis and dermis. The bar represents 10 μm.

Because FAF is a surface protein, we speculated that it might function as an adhesin, mediating bacterial interactions with skin tissue. Thus, bacterial cultures of ALB8 and 505 were incubated with human skin biopsies. Following a washing step the biopsies were incubated for 48 h at 37°C under strict anaerobic conditions allowing bacterial growth. Scanning electron microscopy (SEM) demonstrated that ALB8 bacteria adhere to the basement membrane between epidermis and dermis (Fig. 5A), whereas 505 bacteria did not adhere to the tissue (Fig. 5B).

**Fig. 6:**
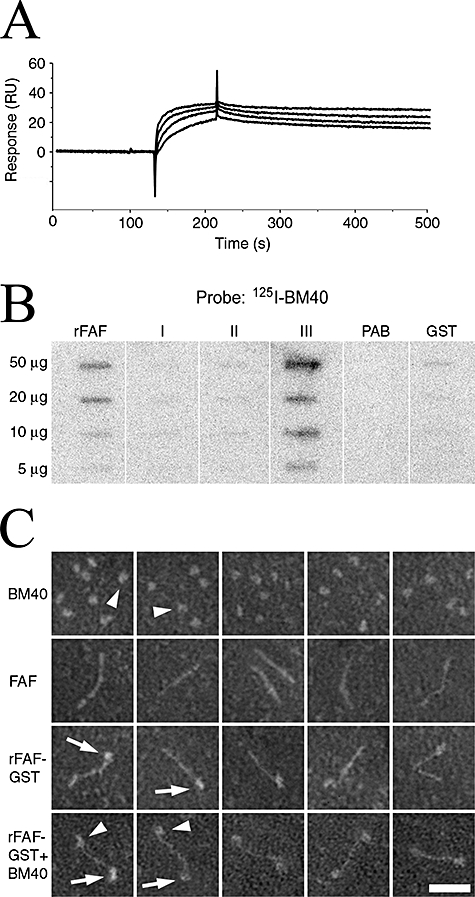
FAF interacts with BM-40. A. Surface plasmon resonance spectroscopy showing the affinity between the basement membrane component BM-40 and FAF. BM-40 was immobilized onto a CM5 sensorchip, and protein FAF was injected at concentrations from 31 to 250 nM at a flow rate of 50 μl min^−1^. B. Intact FAF, fragments of FAF (see Fig. 2C), protein PAB and GST were applied onto PVDF membranes. Membranes were probed with ^125^I-labelled BM-40, and binding was detected using the Fuji Imaging System. C. Electron micrographs after negative staining of FAF in complex with BM-40. The globular N-terminal GST-tag of recombinant FAF is indicated by arrows. BM-40 binds to the C-terminal part of FAF (arrowheads). At the concentrations used (10 nM) no FAF dimer formation was observed. The bar represents 25 nm.

Basement membranes are mainly formed by independent networks of collagen IV and laminin, linked by other molecules, such as nidogen and BM-40 (for a review see Timpl, 1996). To analyse a possible interaction between FAF and various protein components of basement membranes, plasmon surface spectroscopy was utilized. The nine proteins (see *Experimental procedures*) were immobilized, and FAF was applied and left to interact to the level of saturation. Of the tested proteins, only BM-40 showed affinity for protein FAF (Fig. 6A). Association and dissociation constants for the interaction were calculated [7.5 × 10^8^ (1/M) and 1.3 × 10^−9^ (M) respectively] and demonstrated a high affinity for BM-40. Protein PAB had no affinity for any of the proteins. Using the various FAF fragments in a slot binding assay with radiolabelled BM-40 as the probe, the binding site for BM-40 on protein FAF was localized to the C-terminal part of the molecule (Fig. 6B). Again, there was no interaction between protein PAB and BM-40. Moreover, the material solubilized with CNBr from the FAF-expressing strains (see Fig. 4) bound radiolabelled BM-40 in slot blot experiments (data not shown). Next, FAF–BM-40 complexes formed in solution were analysed by electron microscopy following negative staining. The results confirm that the C-terminal part of FAF is the major binding region for BM-40 (Fig. 6C). Most likely, this binding does not compete with FAF self-association. Thus, when radiolabelled BM-40 together with a 1000-fold molar excess amount of unlabelled FAF was incubated with FAF immobilized on a PVDF membrane, the binding of BM-40 was not affected (data not shown).

### FAF-expressing *F. magna* colocalizes with BM-40 in human skin

**Fig. 7:**
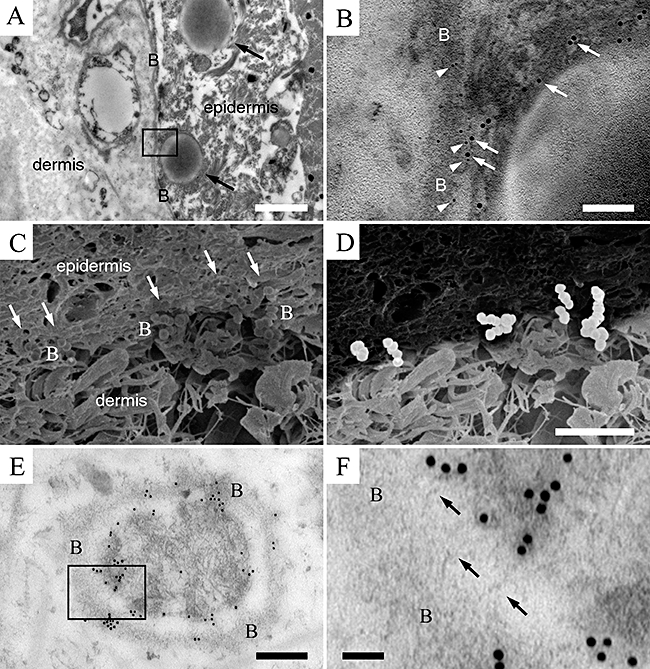
FAF-expressing *F. magna* bacteria are found at the basement membrane in human epidermis and colocalize with BM-40. Human skin biopsies were anaerobically incubated with *F. magna* ALB8 bacteria for 48 h at 37°C and prepared for ultrathin sectioning/transmission electron microscopy. A. An overview showing the epidermis, the basement membrane at the epidermal/dermal junction (B) and dermis. Arrows point at ALB8 bacteria. The bar represents 1 μm. B. The square in A indicates the selected area shown at higher magnification here. FAF-expressing ALB8 bacteria colocalize with BM-40 at the basement membrane (B). Arrows point at anti-FAF antibodies (10 nm gold) and arrowheads point at anti-BM-40 antibodies (5 nm gold). The bar represents 100 nm. C. Scanning electron micrograph of a thin section of a skin biopsy from a healthy donor. Colonies of cocci (arrows) are found at the basement membrane (B) at the epidermal/dermal junction. D. Interpretation of C in pseudocolours. The scale bar represents 10 μm. E. Ultrathin sectioning and transmission electron microscopy of a skin biopsy from a healthy donor after immuno-labelling of protein FAF. Bacteria labelled with colloidal gold are seen at the basement membrane (B). The bar represents 100 nm. F. A selected area indicated by the square in E at higher resolution is shown. Fibrillar protrusions labelled with colloidal gold (arrows) extend from the bacterial surface and interact with the basement membrane (B). The bar represents 50 nm.

To investigate whether FAF mediates adhesion of *F. magna* to skin tissue via the interaction with BM-40, colocalization studies were performed. Human skin biopsies were incubated with ALB8 bacteria for 1 h, and following extensive washing bacteria were allowed to multiply anaerobically for 48 h. The biopsies were then incubated with antibodies against FAF and BM-40 labelled with colloidal gold, 10 and 5 nm respectively. Analysis by electron microscopy shows bacterial cells in the epidermal compartment close to the basement membrane (Fig. 7A). Immuno-staining identifies FAF on the bacterial surface, and BM-40 present in the basement membrane colocalizes with protein FAF (Fig. 7B).

Next, we investigated if endogenous *F. magna* could be identified in human skin. SEM of skin biopsies identified colonies of cocci at the epidermal/dermal junction (Fig. 7C and D). Following immuno-labelling with gold-labelled antibodies against FAF, thin sections of skin biopsies were analysed by electron microscopy. The detection of immuno-labelled bacteria in skin biopsies demonstrates the presence of FAF *in vivo* (Fig. 7E and F), and in summary the results demonstrate that *F. magna* colonizes human skin through the interaction between FAF and BM-40.

### The antibacterial activity of LL-37 is blocked with protein FAF

**Fig. 8:**
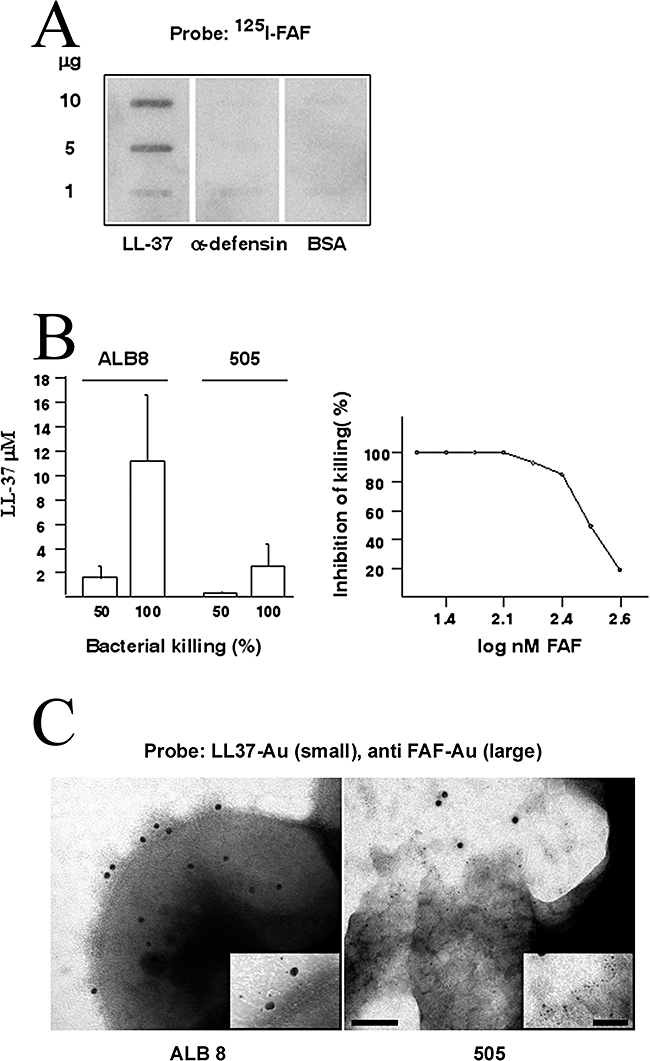
Protein FAF interferes with the antibacterial activity of LL-37. A. Various amounts of the antibacterial peptides LL-37 and α-defensin and BSA were applied to a PVDF membrane. The membrane was incubated with ^125^I-labelled FAF, and binding was detected using the Fuji Imaging System. B. Left panel: *F. magna* strains ALB8 and 505 (2 × 10^6^ cfu ml^−1^) were incubated with the antibacterial peptide LL-37 at various concentrations for 1 h at 37°C, and the cfu were determined. The bars represent the mean ± standard error of the mean of at least three experiments. Right panel: The bactericidal activity of LL-37 at 2.2 μM against 505 bacteria was inhibited with recombinant FAF. Experiments were repeated at least three times and a representative experiment is shown. C. ALB8 and 505 bacteria were grown under strict anaerobic conditions at 37°C for 3 days in TH broth supplemented with 0.5% Tween-80. Bacteria were washed and incubated with LL-37 and anti-FAF antibodies labelled with colloidal gold for 30 min at room temperature (LL-37, 5 nm gold; anti-FAF IgG, 20 nm gold). Following negative staining, samples were analysed by electron microscopy. The bar represents 100 nm. A selected area indicated by the square is shown at higher resolution; bar represents 50 nm.

To successfully colonize its host bacteria needs to overcome innate defence mechanisms, such as antibacterial peptides. Cathelicidins and defensins are two major families of antibacterial peptides having broad bactericidal activity against Gram-positive and Gram-negative bacteria (for references, see Bevins, 2003; Boman, 2003). When LL-37 (a cathelicidin) and α-defensin were applied to a PVDF membrane, radiolabelled FAF was found to bind LL-37 but not α-defensin (Fig. 8A). Using the various FAF fragments as probes, it was demonstrated that regions in both the N- and C-terminal regions are required for full binding of LL-37 (not shown).

The bactericidal effect of LL-37 was tested against *F. magna* strains ALB8 and 505. Significantly higher concentrations of LL-37 were required to kill the FAF-expressing strain ALB8 as compared with the non-expressing 505 strain (Fig. 8B). As protein FAF is also shedded from the bacterial surface (see Fig. 2A, lane 2), we hypothesized that soluble FAF could block the activity of LL-37. In these experiments we used 505 bacteria. The inhibitory effect of recombinantly expressed protein FAF was tested at a bactericidal concentration (2.2 μM) of LL-37, and as shown in Fig. 8B, FAF dose-dependently blocked the killing by LL-37. The ALB8 and 505 strains were also incubated with gold-labelled LL-37 (5 nm gold) and anti-FAF antibodies (20 nm gold), and following negative staining bacteria were analysed by electron microscopy. The anti-FAF antibodies bound to the hair-like projections protruding from the surface of ALB8 bacteria, and LL-37 bound to these projections (Fig. 8C). ALB8 bacteria appeared intact, whereas ejected cytoplasmic material was observed in 505 bacteria indicating a disintegrated cell membrane. LL-37 was also found at the bacterial cell membrane of these bacteria and within ejected membrane blebs (Fig. 8C). These results suggest that protein FAF at the bacterial surface and in solution protects *F. magna* from the bactericidal effect of LL-37. In addition, recombinant FAF inhibited the bactericidal activity of LL-37 (0.9 μM) against group G streptococci, *S. aureus* and *E. coli* (data not shown), further emphasizing the inhibitory effect of FAF on this antibacterial peptide.

## Discussion

Our current understanding of the biology of the anaerobic commensal microbiota is limited despite the fact that a large number of anaerobic bacterial species colonize all non-sterile body surfaces, and many questions concerning fundamental prerequisites for the successful colonization of the human host by these bacteria remain unanswered. Where in the tissue are they located? What is the molecular basis for adherence and colonization, and how do they cope with host defences, including antibacterial peptides? These defence mechanisms should primarily be directed against pathogens but will presumably have a profound impact also on members of the normal microbiota. To address these questions is relevant from a general biological point of view, but it is also of interest in relation to infection medicine. The host–pathogen relationship represents a delicate balance, and modern medicine is gradually increasing the pathogenic potential and significance of commensals. The wide-spread use of antibiotics causes disturbances in the ecology of the normal microbiota. In addition, a growing elderly population, the introduction of foreign materials and devices (prosthetic heart valves, joint replacements, catheters, etc.) and a rapidly expanding number of individuals treated with immunosuppressive drugs have created a situation where the clinical significance of opportunistic pathogens, including anaerobes, has increased dramatically during the last decades.

*Finegoldia magna*, the subject of the present investigation, is a major constituent of the normal anaerobic microbiota, and previous studies have also demonstrated its growing importance as a pathogen (for references see Murdoch, 1998). During cultivation of *F. magna* we noticed that a majority of the isolates formed large bacterial aggregates and that the culture supernatants sometimes contained visible proteinaceous precipitates. These observations initiated the present study and led to the discovery of protein FAF. FAF was found to mediate bacterial aggregation through protein–protein interactions between FAF molecules on neighbouring *F. magna* bacteria. Presumably, these interactions will play a role also *in vivo* by establishing contact between bacteria and facilitating adherence and colonization. The α-helical coiled-coil structure of FAF explains the elongated fibrous appearance of the protein. It is noteworthy that M proteins of the human pathogen *S. pyogenes* (for references see Fischetti, 1989) are also α-helical coiled-coil surface proteins (Phillips *et al*., 1981) that promote bacterial aggregation (Frick *et al*., 2000). FAF and M proteins show no sequence homology, but obviously their related overall structure and localization have similar functional consequences. Moreover, FAF and M proteins are also both released from the bacterial surface. In the case of M proteins, proteolytic enzymes, secreted by *S. pyogenes* or by activated neutrophils, solubilize surface-associated M protein into the environment (Berge and Björck, 1995; Herwald *et al*., 2004). Interestingly, *F. magna* secretes a subtilisin-like proteinase, SufA (Karlsson *et al*., 2007), and ongoing work in our laboratory has demonstrated that this enzyme efficiently releases a 53 kDa FAF fragment similar to the fragment released by CNBr described here (Fig. 2). Like the M proteins that are classical virulence determinants of *S. pyogenes* interacting with several host molecules, such as fibrinogen, albumin, plasminogen and IgG (for references see Cunningham, 2000), FAF could be important for the pathogenesis of *F. magna*.

Prior to this study, no data have been published describing the precise localization of *F. magna* in a human tissue. In normal healthy skin, electron microscopy and FAF antibodies identified *F. magna* at the basement membrane at the epidermal/dermal junction. Thanks to a generous gift from the late Dr Rupert Timpl, we could analyse a unique collection of highly purified basement membrane proteins and their interactions with FAF. These studies revealed that BM-40, a non-collagenous glycoprotein (also called SPARC or osteonectin), is the ligand for FAF. Originally isolated from bone (Termine *et al.*, 1981), BM-40 has later been identified in numerous other tissues (for a review see Brekken and Sage, 2000), and the protein is present extracellularly both in the epidermal and in the dermal compartments of the skin. In FAF, the binding site for BM-40 is in the C-terminal part of the protein. In contrast to the variable N-terminal, the C-terminal part is conserved among FAF homologues, suggesting an evolutionary pressure to preserve this region and underlining the biological significance of the FAF–BM-40 interaction. Apart from skin, *F. magna* also colonizes other epithelial surfaces. The fact that there are *F. magna* isolates that do not carry the *faf* gene indicates that strains in other ecological niches utilize other mechanisms than FAF–BM-40 interactions for colonization.

Most of the FAF-expressing strains of this study (20 of 28) also express the albumin-binding PAB protein (de Château and Björck, 1994). Previous work has demonstrated that the binding of albumin to *F. magna* promotes their growth (de Château *et al*., 1996), and it is interesting that BM-40 was reported to increase albumin transport across the endothelium (Goldblum *et al*., 1994). Hypothetically, the FAF–BM-40 interaction could influence this transport and thereby bacterial multiplication. *F. magna* is among the most frequently isolated bacterial species from chronic leg ulcers (Wall *et al*., 2002; Stephens *et al*., 2003). *In vitro*, growth medium from such *F. magna* isolates inhibits the proliferation of fibroblasts and keratinocytes (Stephens *et al*., 2003). Soluble BM-40 is found in wound fluid and was reported to stimulate wound healing, angiogenesis and cell proliferation (for references see review Brekken and Sage, 2000). Moreover, an increased production of BM-40 is induced in fibroblasts by transforming growth factor β1 produced in remodulating tissue (Wrana *et al*., 1991). Through the interaction with BM-40, FAF-expressing *F. magna* and/or soluble FAF may impair wound healing and explain why these bacteria so commonly colonize chronic wounds.

In epidermis and on other epithelial surfaces a soluble barrier of antimicrobial peptides (AMPs) provide a non-specific first line of defence against potential pathogens (Zasloff, 2002). In case of injury and infection, AMPs are upregulated in the skin as a result of increased synthesis by keratinocytes and degranulation of neutrophils (Braff *et al*., 2004). The AMP LL-37/hCAP18 is induced in keratinocytes during inflammation (Frohm *et al*., 1997; Dorschner *et al*., 2001), while human neutrophil α-defensins (HNP1-HNP3) are expressed also by monocytes and natural killer cells (for references see Selsted and Ouellette, 2005). A key observation of this study is that FAF-expressing *F. magna* is more resistant to LL-37 and that soluble FAF also binds and inactivates LL-37. FAF showed no affinity for the α-defensin HNP1, and it is interesting that this AMP does not kill *F. magna* (Karlsson *et al*., 2007). Large amounts of FAF are released by *F. magna* growing *in vitro* (Fig. 2A, lane 2) and also presumably *in vivo*. In the tissue, such released material could form protective barriers surrounding colonies of *F. magna*.

Opportunistic bacterial infections represent a major and growing medical problem. To address this problem, a better understanding of the molecular and cellular interplay between commensal bacteria and their host is required. The properties of FAF demonstrate that the protein significantly influence this interplay and help to explain how *F. magna* colonizes and survives in its human host.

## Experimental procedures

### Bacteria, growth conditions, analysis of bacterial aggregation and CNBr treatment

*Finegoldia magna* strains (30) were isolated at the Department of Clinical Microbiology, Lund University Hospital, Sweden. All strains except two that were normal skin isolates were isolated from patients with various pathological conditions, mostly infected skin wounds (12). The FAF-expressing strains that were sequenced were isolated from the following anatomical sites: scrotal abscess (ALB8), intra-abdominal abscess (L3410), knee infection (1766), foot wound (1462), foot abscess (2133), normal skin (ELTI). Other sites included liver, sinus, urethra and cervix. The 505 strain was isolated from urethra. The bacteria were grown under strict anaerobic conditions in Todd-Hewitt broth (TH) (Difco) supplemented with 0.5% Tween-80 at 37°C. Group G streptococci and *S. aureus* were cultivated in TH at 37°C, and *E. coli* was grown in Luria–Bertani broth at 37°C. For analysis of bacterial aggregation *F. magna* strains were grown for 3 days in 10 ml test tubes. The bacteria were gently resuspended and left to settle at 4°C, and the degree of aggregation was followed by measuring the optical density at 620 nm in the upper half of the tubes. ALB8 bacteria, washed with PBS (0.12 M NaCl, 0.03 M phosphate, pH 7.4), were also analysed for aggregation in presence of anti-FAF antibodies. Three millilitres bacterial solution [1 × 10^9^ colony-forming units (cfu) ml^−1^] were mixed with anti-FAF IgG (0.54 mg), and sedimentation was measured as above. CNBr extraction of *F. magna* surface proteins was performed as previously described (Otten *et al*., 1992). CNBr-treated ALB8 bacteria were washed with PBS and tested for aggregation. A portion of the cells was subjected to negative staining, see below.

### Proteins, peptides, coupling of proteins to Sepharose, antibodies, labelling of proteins and binding assay

Human and bovine serum albumin (HSA and BSA) were purchased from Sigma, and recombinant protein PAB was prepared as described previously (de Château and Björck, 1994). The LL-37 peptide was synthesized by Innovagen and human neutrophil protein 1 was from Bachem. Basement membrane proteins (Laminin-1/Nidogen-1, Nidogen-1, Nidogen-2, Collagen IV, BM-40, Fibulin-1C, Fibulin-2, Laminin α1 and Laminin α2) were kindly provided by the late Dr Rupert Timpl. The monoclonal antibody against bovine osteonectin/BM-40, developed by Dr John D. Termine, was obtained from the Developmental Studies Hybridoma Bank developed under the auspices of the NICHD and maintained by the Department of Biological Sciencies, University of Iowa, Iowa City, USA. Coupling of HSA to CNBr-activated Sepharose 4B (Pharmacia) was performed according to the manufacturer's instructions. Purification of protein FAF shedded from *F. magna* strain ALB8 was performed as follows. Bacterial cells were washed with PBS, subjected to vortexing for 60 s, followed by centrifugation at 3000 × *g*. The supernatant was collected and incubated with HSA-Sepharose for 3 h at 4°C. Unbound material, devoid of the HSA-binding protein PAB, was collected and left at 4°C. The precipitated material was dissolved in PBS and subjected to dialysis against the same buffer. Antibodies against recombinant protein FAF were raised in rabbits, and anti-protein FAF IgG was purified on a protein G-Sepharose column (Amersham Bioscience). Anti-FAF antiserum was applied to the column, and following extensive washing with PBS bound IgG was eluted with 0.1 M glycine-HCl, pH 2.0. Eluted IgG was dialysed against PBS. Recombinant protein FAF, fragments of FAF, HSA and human BM-40 were radiolabelled with ^125^I using IODO-BEADS® Iodination reagent (Pierce). Binding of ^125^I-labelled proteins to *F. magna* bacteria was performed as described earlier (Björck and Kronvall, 1984).

### Electrophoresis, Western blot analysis and analysis of proteins applied to PVDF membranes

The SDS-PAGE was performed using the buffer system by Laemmli. The polyacrylamide content was 10% in the separation gels with a cross-linking of 2.7%. Samples were boiled for 3 min in an equal volume of sample buffer containing 2% SDS and 5% 2-mercaptoethanol. Gels were stained with Coomassie brilliant blue or transferred to PVDF membranes (Immobilon, Millipore) for Western blot analysis. Proteins were also directly applied to PVDF membranes using a Milliblot-D system (Millipore). The membranes were blocked, incubated with ^125^I-labelled proteins and washed as previously described (Åkesson *et al*., 1990). Bound ligand was detected using the Fuji Imaging System. Alternatively, membranes were blocked with PBS containing 0.05% Tween-20 (PBST) and 5% dry milk powder (blocking buffer), incubated with antibodies against FAF (1:1000) in blocking buffer for 30 min at 37°C, washed with PBST and incubated with peroxidase-conjugated protein A (Sigma) 1:3000 in blocking buffer for 30 min at 37°C. The membranes were washed with PBST, and bound antibodies were detected using the chemiluminescence method.

### Cloning, polymerase chain reactions, sequencing techniques and expression of protein FAF

For cloning and sequencing of the gene encoding FAF the 5′ primer (3209) 5′-GCA(T)GAAAAA(G)GCA(T)CCA(TC)AAA(G)AAT(CA)ACNGAAAAT(C)CT-3′ and the 3′ primer (769) 5′-GCTACCAGCTTTTGGTAA-3′ were used in PCR with chromosomal DNA from *F. magna* strain ALB8. A fragment of 1.7 kb was generated, cloned and sequenced (see below). Two primers, based on the sequence retrieved from the 1.7 kb product, were synthesized to obtain the signal sequence and the terminal 3′-end of *faf*. The 5′ primer (4244) 5′-GTTATCGACAAAGGAACTATCG-3′ from the 3′-end of the 1.7 kb sequence and the 3′primer (4292) 5′-CAGAAGCCCATGGTTGAGCTAC-3′ from the 5′-end of the sequence were used in PCR. As template a ligation mix of HindIII-cleaved genomic DNA from *F. magna* strain ALB8 and a HindIII-cleaved 450 bp PCR fragment generated with genomic DNA from *F. magna* strain KA were used. The 450 bp product was generated with the 5′ primer (KAN1) 5′-GTCACATGTTAGCAGTAACAAC-3′ and the 3′ primer (KAC1) 5′-GAAGTTGATGAGTCACCATTCATG-3′. This KA fragment contains a HindIII site, and the fragments obtained after cleavage were used as adaptors to the HindIII-cleaved fragments from ALB8. Primers KAN1 and 4292 generated a 0.6 kb PCR fragment and primers 4244 and KAC1, a fragment of 0.48 kb. The PCR products were cloned into the T/A-vector pGEM-T (Promega) according to the instructions from the manufacturer. Positive clones were sequenced.

For expression of the mature protein FAF (a.a. 28–616) in *E. coli*, *faf* was PCR amplified using the 5′ primer (faf1) 5′-GCAGAATTCGCAGAAAAAGCCCCAAAGATTACTGAAA-3′, containing an EcoRI site and the 3′ reverse primer (faf2), containing a NotI site, 5′-ATAGCGGCCGCTTATTATTTACGTTTTTTAAGTGAAATA-3′. Fragments of FAF were expressed using the following primers: for fragment I (a.a. 28–115) and fragment II (28–317) the 5′ primer faf1 in combination with the 3′ reverse primers 5′-ATAGCGGCCGCTTATTAAGCTTCTTTGATTGCTTTGTA-3′ for fragment I and 5′-ATAGCGGCCGCTTATTATTCTTTAGATGCTGCATCGAA-3′ for fragment II containing a NotI site. For expression of fragment III (a.a. 239–616) the 5′ primer, containing an EcoRI site, 5′-GCAGAATTCGGTGGATGGAATGGATCTGCA-3′, was used together with the 3′ reverse primer faf2. The DNA fragments were cloned into pGEX-6p-1 (Amersham Pharmacia). The FAF constructs, fused to GST, were expressed and purified on glutathione Sepharose according to the manufacturer's instructions. Where required the GST-tag was cleaved off using PreScission protease (Amersham Pharmacia).

To investigate the occurrence of *faf* in *F. magna* isolates, PCR was performed using the following primers: faf1 and faf2, generating a 1700 bp fragment corresponding to the mature protein FAF; the 5′ primer (fafNdown) 5′-GAAACAGCAAAGAAAAAAGCTTAT-3′ hybridizing with nucleotides 377–400 and the 3′ reverse primer (3downfaf) 5′-CTTTTTAGTATAATCTATTTGCG-3′ hybridizing with nucleotides 1977–98 in the *faf* gene; the 5′ primer (signal) 5′-ATGAAATTAAACAAAAAATTATTGAC-3′ hybridizing with nucleotides 121–146 and the 3′ reverse primers (faf3) 5′-CAGTAACTTTGTATGCTCTTTGG-3′ hybridizing with nucleotides 1086–1108, or (faf4) 5′-CGATGTCTCCGTTTTCATCAG-3′ hybridizing with nucleotides 1469–1489 in the *faf* gene. Thirty isolates were analysed and fragments were generated as follows: faf1/faf2 in 13.8%, fafNdown/3downfaf in 27.6%, signal/faf3 in 93.3%, signal/faf4 in 75.9% and signal/3downfaf in 93.3% of the strains. The PCR products from strains L3410, 1462, 2133, ELTI and 1766 were purified with a High Pure PCR purification kit (Roche Applied Science) and used as templates in sequencing reactions using an ABI PRISM® BigDye™ dideoxy terminator kit (BigDye terminator version 3.1 cycle sequencing Ready Reaction, Applied Biosystem), according to the manufacturer's instructions. The nucleotide sequences have been submitted to the GenBank/NCBI under accession numbers: AY192570 strain ALB8, EF577478 strain L3410, EF577479 strain 1462, EF577480 strain 2133, EF577481 strain ELTI, EF577482 strain 1766. Protein domains were identified using the InterProScan program (http://www.ebi.ac.uk/Tools/InterProScan).

### Amino acid sequence analysis, CD spectroscopy and computational sequence analysis

Samples subjected to N-terminal amino acid sequencing were separated by SDS-PAGE, transferred to a PVDF membrane, stained with Coomassie brilliant blue, and the protein bands were cut out from the membrane. Sequence analyses were performed at Eurosequence (the Netherlands). CD spectra of recombinant protein FAF were recorded on a Jasco J-720 spectropolarimeter equipped with a thermostated cell holder. The spectra were recorded in the far UV region (260–185 nm) in cells with path lengths of 0.02 cm. The buffer used in the experiments was 10 mM phosphate buffer, pH 7.5, and spectra were recorded for protein concentrations of 0.1, 0.2 and 0.3 mg ml^−1^ respectively. Coiled-coil predictions were calculated by the program Macstripe, using a 28-residue window, as previously described (Nilson *et al*., 1995). The blast Network server at the National Center for Biotechnology Information was used for similarity searches (Altschul *et al*., 1990).

### Surface plasmon resonance spectroscopy

The different basement membrane components were diluted with 10 mM sodium acetate, pH 4.0 and immobilized via amine coupling in different flow cells of a CM5 sensorchip (BIAcore, Uppsala, Sweden). Immobilization levels were around 1500 resonance units. For affinity measurements, binding and dissociation were monitored in a BIAcore 2000 instrument. Proteins FAF and PAB were injected at concentrations ranging from 31 nM to 250 nM, over the coated surfaces at 50 μl min^−1^ and 25°C (in running buffer: 10 mM Hepes, pH 7.5, 150 mM NaCl, 0.005% surfactant P20 and 3.4 mM EDTA). A flow cell subjected to the coupling reaction without protein was used as a control for bulk resonance changes. The basement membrane surfaces were regenerated by injection of a 200 μl pulse of running buffer containing 2 M NaCl followed by an extensive wash. After X and Y normalization, the blank curves from the control flow cell of each concentration were subtracted, and association (*k*_*a*_) and dissociation (*kDa*) rate constants were determined using a Langmuir fit in the BIA Evaluation 3.0 program. The equilibrium dissociation constants (*K*_*D*_) were calculated from these values.

### Bactericidal assay

*Finegoldia magna* strains ALB8 and 505 were grown to mid-log phase (A_620_ ≈ 0.4–0.6) in TH broth (supplemented with 0.5% Tween-80), washed and diluted in 10 mM Tris-HCl, pH 7.5, containing 5 mM glucose. 50 μl of bacteria (2 × 10^6^ cfu ml^−1^) was anaerobically incubated with LL-37 at various concentrations for 1 h at 37°C. To quantify the bactericidal activity serial dilutions of the incubation mixtures were plated on TH agar, incubated anaerobically for up to 3 days, and the number of cfu was determined. In subsequent experiments, 505 bacteria were incubated for 1 h with LL-37 at a concentration of 2.2 μM together with different amounts of protein FAF. *S. aureus*, group G streptococci and *E. coli*, respectively, were incubated with LL-37 at a concentration of 0.9 μM together with different amounts of protein FAF, and the bactericidal activity was quantified.

### Adherence to human skin

Punch biopsies (4 mm diameter) of human skin were obtained in connection with skin transplant surgery. Informed consent was obtained from the patients. The Ethics Committee at Lund University approved the use of this material (permit numbers LU 509–01 and LU 708–01). The biopsies were incubated with *F. magna* bacteria (2 × 10^8^ bacterial cells) in PBS for 1 h at room temperature and washed with PBS to remove non-adherent bacteria. Minimal essential medium (MEM; Life technologies) was added, and the samples were incubated anaerobically at 37°C for 48 h. Following a washing step with PBST samples were fixed in 2.5% glutaraldehyde in 0.15 M sodium cacodylate, pH 7.4 and prepared for SEM (see below). For colocalization experiments antibodies were gold-labelled as described (Baschong and Wrigley, 1990): anti-protein FAF (10 nm gold) and anti-BM-40 (5 nm gold). Biopsies were incubated and washed as above, incubated with gold-labelled antibodies, washed and prepared for ultrathin sectioning and transmission electron microscopy. In some experiments, biopsies were subjected to low temperature embedding in Lowicryl, ultrathin sectioning and transmission electron microscopy without prior incubation with *F. magna* bacteria.

### Electron microscopy

For SEM 20 μl of bacterial cultures, containing 2 × 10^9^ bacterial cells ml^−1^ in PBS, was added on top of a poly L-lysine-coated glass coverslip for 30 min. Subsequently specimens were fixed in 2.5% glutaraldehyde in 0.15 M sodium cacodylate, pH 7.4 for 2 h at room temperature, washed and stored in 0.15 M cacodylate buffer, pH 7.4. Fixed specimens were dehydrated for 10 min at each step of an ascending ethanol series and inserted into a Balzers critical point dryer using 100% ethanol as the intermediate solvent. The pressure chamber was then extensively flushed three times with carbon dioxide to remove all traces of residual ethanol. The samples were critical point dried, mounted on aluminium holders, palladium/gold sputtered and examined in a Jeol ST300 SEM.

For transmission electron microscopy bacterial cells were collected by centrifugation at 3000 × *g*, fixed for 1 h at room temperature and then overnight at 4°C in 2.5% glutaraldehyde in cacodylate buffer. Samples were washed with cacodylate buffer, post-fixed for 1 h at room temperature in 1% osmium tetroxide in cacodylate buffer, dehydrated in a graded series of ethanol and then embedded in Epon 812 using acetone as intermediate solvent. Specimens were sectioned with a diamond knife into 50 nm-thick ultrathin sections on an LKB ultramicrotome. The ultrathin sections were stained with uranyl acetate and lead citrate. Specimens were observed in a Jeol JEM 1230 electron microscope operated at 80 kV accelerating voltage, and images were recorded with a Gatan Multiscan 791 CCD camera.

The following samples were prepared for negative staining: extract of ALB8 bacteria heated at 80°C for 10 min, recombinant protein FAF (0.1 mg ml^−1^), complexes of protein FAF and BM-40 preformed by incubation of equimolar concentrations (2 nM) for 20 min at room temperature, untreated and CNBr-treated ALB8 bacteria (100 × 10^6^ cfu) washed with TBS (50 mM Tris-HCl, pH 7.5, 0.15 M NaCl), ALB8 and 505 bacteria (100 × 10^6^ cfu) incubated with gold-labelled LL-37 (5 ng; 5 nm gold) and anti-FAF antibodies (20 nm gold) for 30 min at room temperature. Samples were adsorbed onto a 400-mesh carbon-coated copper grid, which was rendered hydrophilic by glow discharge at low pressure in air. The grid was immediately blotted, briefly washed with two drops of water and stained with 0.75% uranyl formate for 15 s. Specimens were studied in a Jeol 1200 EX transmission electron microscope operated at 60-kV accelerating voltage and ×75 000 magnification.
