# Supplementary material for: Identification of a novel protein promoting the colonization and survival of Finegoldia magna, a bacterial commensal and opportunistic pathogen
Source: Mol Microbiol. 2008 Sep 25;70(3):695–708. doi: 10.1111/j.1365-2958.2008.06439.x (PMC2628433; doi:10.1111/j.1365-2958.2008.06439.x)
Supplement: Supplementary file 1 [file mmi0070-0695-SD1.pdf]

Supporting Material

Identification of a novel protein promoting the colonization and survival of *Finegoldia magna*, a bacterial commensal and opportunistic pathogen

Inga-Maria Frick, Christofer Karlsson, Matthias Mörgelin1, Anders I. Olin, Radmila Janjusevic, Clara Hammarström, Elisabet Holst, Maarten de Château, and Lars Björck

Fig. S1. Sequence alignment of protein FAF from *F. magna* strains. The *faf* gene was sequenced from strains L3410, 1462, 2133, ELTI and 1766 (GenBank accession numbers EF577478, EF577479, EF577480, EF577481, EF577482, respectively), and the derived amino acid sequences were compared with the sequence of FAF from the ALB8 strain (accession number AY192570). The sequence of the FAF homologue present in the recently reported genome sequence of *F. magna* ATCC 29328 strain is also included (accession number BAG08753).

Fig. S1

Alignments protein FAF

```
                    10        20        30        40        50        60        70        80
ALB8        MKLNKKLLTAALAGALIVTAVPANTFAA--EKAPKITEN--LSEEQAAAKIAELSAEIKTLSERRAEIVAQPWASELTQE
L3410       MKLNKKLLTAALAGALIVTAVPANTFAA--EKAPKITEN--LSEEQAAAKIAELSAEIKTLSERRAEIVAQPWASELTQE
1462        MKLNKKLLTAALAGALVVTAVPAGTFAEKNSKENSYTEELNLTKQQAAARMDQLAKKIAELSARRAAIVTQEWKAGETSE
2133        MKLNKKLLTAALAGALVVTAVPAGTFAEKNSKENSYTEELNLTKQQAAARMDQLAKKIAELSARRAAIVTQEWKAGETSE
ELTI        MKLNKKLLTAALAGALVVTAVPAGTFAEKNSKENSYTEELNLTKQQAAARMDQLAKKIAELSARRAAIVTQEWKAGETSE
1766        MKLNKKLLTAALAGALIVTAVPASTFADYNKAEESYTYLLNLSKEQAAAKIAEFTAIIKEKSARRAKIVAQPFNPDGTSE
ATCC 29328  MKLNKKLLTAALAGALIVTAVPAGTFAK--EAEVNTNTNKYLTAEQAAAKVKKLSEEINDLSARRKSIVAQAYKIGETAE

                    90       100       110       120       130       140       150       160
ALB8        GIIEKLQAEETAKKKAYEGAKAAYESATKAWEAAKDAQEKARLAKIEADKKAKAAQGDYYKAIKEAEKERDAAKKAAKEA
L3410       GIIEKLQAEETAKKKAYEGAKAAYESATKAWEAAKDAQEKARLAKIEADKKAKAAQGDYYKAIKEAEKERDAAKKAAKEA
1462        GLIEKLQKEEAAAKKAYEAAKANFEAAAKAWEAAKDAQEKARLAKRKALDAYDAKVGEYNRLVKEAEAARDAAKKEAKEK
2133        GLIEKLQKEEAAAKKAYEAAKANFEAAAKAWEAAKDAQEKARLAKRKALDAYDAKVGEYNRLVKEAEAARDAAKKEAKEK
ELTI        GLIEKLQKEEAAAKKAYEAAKANFEAAAKAWEQAKDAQEKARLAKRKALDAYDAKVGEYNKLVKEAEAARDAAKKEAKEK
1766        GLIEKLQQEEAKAKKAYEAAKATYEAAEKALTAAKDNEEKARLNLIKVNDAFDAKYGEYKAAVAKATSEYEAAKEKANRE
ATCC 29328  GTIEVLQRKEMAAKAEYESAKAAFEGAKKAWEAAKDNEEKARLAEIAAKDALAAKKGDYGKLLQAAKDARKAAEEAADRQ

                   170       180       190       200       210       220       230       240
ALB8        YDTTIANLQRKFESATAAADKALIELNSAKEALDSAKKSGSNEE-QLTRLRKEVEEKELAHETAKNLATRASNERADA--
L3410       YDTTIANLQRKFESATAAADKALIELNSAKEALDSAKKSGSNEE-QLTRLRKEVEEKELAHETAKNLATRASNERADA--
1462        FEDTIASANRKYEVAQTNLDRAENELAAAKKALETIADDEDENIPEGTNAQYDQAKKAVRDLQAKVAELEVERAKANDEK
2133        FEDTIASANRKYEVAQTNLDRAENELAAAKKALETIADDEDENIPEGTNAQYDQAKKAVRDLQAKVAELEVERAKANDEK
ELTI        FEDTIASSNRKYEVAQTNLDRAENELAAAKKALETIADDEDENVPEGTKAQYDQAKKAVRDLEAKVAQLKIERTKAHDEK
1766        YETVIANSKRKSEDAKVAYDKTVADKEAAEKALEAAVKAGAEKG-HISELRIIAEQKTQAEAQAKLVY-DAEVAKIDD--
ATCC 29328  YDTTIAGLQRKFETAQTVADEAKIEYNKAVKAYDTAVAEKQNEE-QLTRLRKEKENKKAALDKANNALEAASNERADV--

                   250       260       270       280       290       300       310       320
ALB8        ---KSGAEKALAAAENQADENFQAKKQYLDKKYILSGGWNGSAADLTKLERERLAAIDAYNNAVANTKAAHDAYISARDA
L3410       ---KSGAEKALAAAENQADENFQAKKQYLDKKYILSGGWNGSAADLTKLERERLAAIDAYNNAVANTKAAHDAYISARDA
1462        ITVKNTAKENLEKANKQADTLFQAKLQELNKKYKLNDNYGDAAAELTLIERAKLKAIKDYDNAKANAEAAFNNYVAANKA
2133        ITVKNTAKENLEKANKQADTLFQAKLQELNKKYKLNDNYGDAAAELTLIERAKLKAIKDYDNAKANAEAAFNNYVAANKA
ELTI        ITVKDTAKENLEKANDQADTLFQAKIQELNKKYKLNDNYGDAAAELTLIERAKLKAIKDYDNAKANAEAAFGNYVAANKA
1766        --TVNAAKETQKSAFNTARNAYDAEINRLNKKYNLSDGWNESAAELTKARREIEKATNAYVAAQAGTVSAQKAYDSALEG
ATCC 29328  ---SKSAKETRDKAYEVAANNYTAEKQRLDYIYKLNGNYGDKVAELTKLERARINATNAYEDAKAATKAAHEAYISARDN

                   330       340       350       360       370       380       390       400
ALB8        FDAASKEYAAATQRTYKITEELKSIEKQLKEKFVTINAILKDQNKGINSLADQLNSELIKELEEFSKLSSSEQFAKIAKL
L3410       FDAASKEYAAATQRTYKITEELKSIEKQLKEKFVTINAILKDQNKGINSLADQLNSELIKELEEFSKLSSSEQFAKIAKL
1462        YEAASKAYAKANQRAYKVTEELKSIEKQLKENYVRINIILKDQNQGINSLAGKLDADMIKEIENFAELSKSEQAAAIAKL
2133        YEAASKAYAKANQRAYKVTEELKSIEKQLKENYVRINIILKDQNQGINSLAGKLDADMIKEIENFAELSKSEQAAAIAKL
ELTI        YEAASKAYAKANQRAYKVTEELKSIEKQLKENYVRINIILKDQNQGINSLAGKLDSDMIKEIENFAELSKSEQAAAIAKL
1766        YKAAKKAYAAANQRTYKVSEELKTIEKVLKENYTKINVILADQNKSINDLASQLSAETVKAIQDWAKLSGSEQAAQIAKL
ATCC 29328  YDAKSKAYAEANQKTYKVTEELKSIEKQLKEKFVAIKEILNDQNKNINDLADKLNADMIKELEDFSKLSSSEQYAKIAEL

                   410       420       430       440       450       460       470       480
ALB8        QEELAKLKKEYAKTSEGIQQVKKLVTYQFHVKDTEGKGVQGLTLRLANIDDTSKVYTATSDANGLIQFKSMVEGKYSVTV
L3410       QEELAKLKKEYAKTSDGIQQVKKLVPYQFHVKDTEGKGVQGLTLRLANIDDTSKVYTATSDANGLIQFKSMVEGKYSVTV
1462        QKELNKLKEEYDKTKDEIKEVKSVYTYKFAVKDTAGKGVQGLSLTFTNIEDSTKVYVGTSDENGDIVVKGIAEGTYKIAV
2133        QKELNKLKEEYDKTKDEIKEVKSVYTYKFAVKDTAGKGVQGLSLTFTNIEDSTKVYVGTSDENGDIVVKGIAEGTYKIAV
ELTI        QKELNKLKEEYDKTKDEIKEVKSVYTYKFAVKDTAGKGVQGLSLTFTNIEDSTKVYVGTSDENGDIVVKGIAEGAYKIAV
1766        QEELNKIKEEYNKAKDEIKEVKSVYTYKFAVKDTAGKGVQGLTLQFTNIEDTSKVYTATSDENGDIVVNGIVEGTYKIAV
ATCC 29328  QKELAKLKKEYAKTSEGIQQVKKLVTYQFHVKDTEGKGVQGLTLRLANVDDTSKVYTATSDANGLIQFKSMVEGKYTVTV

                   490       500       510       520       530       540       550       560
ALB8        TKVPTGYDVYQDGKKIDSVKALFRAESEVKAENNAEETKK---EETKGNKRVDIPSIIDNTTEGNGEKEEKDNKDEKVID
L3410       TKVPTGYDVYQDGKKIDSVKALFRAESEVKAENNAEETKK---EETKGNKRVDIPSIIDNTTEGNGEKEVKDHKDEKVID
1462        THVPTGYKVVQKGKMVSEVKSLFRPEAEVKAENNAEEAKK---DEAKKDATVEIPEYVTIKGG--KEDKKDEKEDEKVID
2133        THVPTGYKVVQKGKMVSEVKSLFRPEAEVKAENNAEEAKK---DEAKKDATVEIPEYVTIKGG--KEDKKDEKEDEKVID
ELTI        THVPTGYKVVQKGKMVSEVKSLFRPEAEVKAENNVEE--K---EEAKK-SEVVVPEIVVIKGA--KEDKKDDKKDEKVID
1766        TKVPTGYKVVQKGKMVSEVKGLFRPEAEVKAENNVEE--K---EEAKK-SEVVVPEIVVIKGA--KEDKKDDKKDEKVID
ATCC 29328  TKVPTGYDVYQDGKKIDSVKALFRAESEVKAENNAEEAKKDAKKEEKGNKRVDIPSVIDTTTE-NKEESSDKKEDEKVID

                   570       580       590       600       610       620       630       640
ALB8        KATIVVEKKKKDNEDNK--------DNKKPEKKE---TKKPAKPAK---QRLPKAGAAAEAATIAAAAMATMGGAYISLK
L3410       KGTIVVEKKKKDNKDNK--------DNKKPEKKE---TKKPAKPAK---QRLPKAGAAAEAATIAAAAMATMGGAYISLK
1462        GGTIVVEKE-KDNKDNK---KPEKEDNKKPEKQEKENNKKPAKPAKT-KKKLPKAGAAAEAATIAAAAMATMGGAYLSLK
2133        GGTIVVEKE-KDNKDNK---KPEKEDNKKPEKQEKENNKKPAKPAKT-KKKLPKAGAAAEAATIAAAAMATMGGAYLSLK
ELTI        GGTIVVEKE-KDNKDNKDNKKPEKEDNKKPEKEEKENNKKPAKPAKKEKKALPKAGAAAEAATIAAAAMATMGGAYLSLK
1766        GGTIVVEKE-KDNKDNKDNKKPEKEDNKKPEKEEKENNKKPAKPAKKEKKALPKAGAAAEAATIAAAAMATMGGAYLSLK
ATCC 29328  AGTIVVKEK--DKKDNK--------DNKKPEKKE---TKKPATPAKPNKKKLPKAGAAAEAATIAAAAMATMGGAYLSLK

                   650       660       670       680       690       700       710       720
ALB8        KRK--
L3410       KRK--
1462        KRKFT
2133        KRKFT
ELTI        KRK--
1766        KRK--
ATCC 29328  KRK--
```
